# Real-time *In Vivo *Recording of *Arabidopsis* Calcium Signals During Insect Feeding Using a Fluorescent Biosensor

**DOI:** 10.3791/56142

**Published:** 2017-08-15

**Authors:** Thomas R. Vincent, James Canham, Masatsugu Toyota, Marieta Avramova, Sam T. Mugford, Simon Gilroy, Anthony J. Miller, Saskia Hogenhout, Dale Sanders

**Affiliations:** ^1^Department of Metabolic Biology, John Innes Centre, Norwich Research Park; ^2^Department of Botany, University of Wisconsin, Madison; ^3^Department of Biochemistry and Molecular Biology, Saitama University; ^4^Precursory Research for Embryonic Science and Technology (PRESTO), Japan Science and Technology Agency (JST); ^5^Department of Cell and Developmental Biology, John Innes Centre, Norwich Research Park

**Keywords:** Biochemistry, Issue 126, Calcium, aphid, GCaMP, microscopy, insect, GFP

## Abstract

Calcium ions are predicted to be key signaling entities during biotic interactions, with calcium signaling forming an established part of the plant defense response to microbial elicitors and to wounding caused by chewing insects, eliciting systemic calcium signals in plants. However, the role of calcium *in vivo* during biotic stress is still unclear. This protocol describes the use of a genetically-encoded calcium sensor to detect calcium signals in plants during feeding by a hemipteran pest. Hemipterans such as aphids pierce a small number of cells with specialized, elongated sucking mouthparts, making them the ideal tool to study calcium dynamics when a plant is faced with a biotic stress, which is distinct from a wounding response. In addition, fluorescent biosensors are revolutionizing the measurement of signaling molecules *in vivo *in both animals and plants. Expressing a GFP-based calcium biosensor, GCaMP3, in the model plant *Arabidopsis thaliana* allows for the real-time imaging of plant calcium dynamics during insect feeding, with a high spatial and temporal resolution. A repeatable and robust assay has been developed using the fluorescence microscopy of detached GCaMP3 leaves, allowing for the continuous measurement of cytosolic calcium dynamics before, during, and after insect feeding. This reveals a highly-localized rapid calcium elevation around the aphid feeding site that occurs within a few minutes. The protocol can be adapted to other biotic stresses, such as additional insect species, while the use of *Arabidopsis thaliana* allows for the rapid generation of mutants to facilitate the molecular analysis of the phenomenon.

**Figure:**
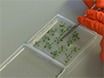


## Introduction

Calcium (Ca^2+^) is one of the most ubiquitous signaling elements in plants. A transient rise in cytosolic Ca^2+^ concentration ([Ca^2+^]_cyt_) is decoded by a complex network of downstream components and is involved in the response to both abiotic and biotic stresses^1^,^2^. A rise in [Ca^2+^]_cyt _is one of the first responses to microbial elicitors, forming a common part of the plant defense response^3^,^4^,^5^. Rises in [Ca^2+^]_cyt_ have also been observed in response to wounding caused by chewing insects, such as lepidopterans^6^,^7^. However, the potential role of plant Ca^2+^ signals in response to live biotic threats that cause damage to only a few cells has not been explored. The green peach aphid *Myzus persicae *is a hemipteran insect that represents a significant threat to world agriculture^8^,^9^, and Ca^2+^ efflux from the extracellular space has been observed in leaves infested with *M. persicae*^10^.This protocol outlines a robust and repeatable method for measuring plant Ca^2+^ signals while *M. persicae* feed from leaves using a fluorescent Ca^2+^ biosensor, with both aphids and GCaMP3 offering novel tools with which to dissect the role of Ca^2+^ during biotic interactions.

Ca^2+^-selective microelectrodes were formerly used to measure [Ca^2+^] in plants^11^,^12^. More recently, bioluminescent and fluorescent approaches have become standardized. These biosensors bind Ca^2+^ and emit light, allowing for un-paralleled opportunities to study Ca^2+ ^dynamics in both cells and whole tissues. Ca^2+^ biosensors can be injected as dyes or stably produced upon the introduction of the biosensor coding sequence into the genome of the organism via transformation (*i.e., *genetically encoded biosensors). The latter offers the major advantages of being easily expressed in live tissue and capable of subcellular localization^13^. The aequorin protein, isolated from *Aequorea victoria* (jellyfish) was the first genetically encoded Ca^2+^ biosensor deployed in plants^14^. As a bioluminescent protein, aequorin does not require excitation by external light, which avoids chromophore bleaching and autofluorescence^15^. Aequorin has been successfully used to measure [Ca^2+^] fluxes in response to various stimuli, including temperature^16^, pathogens^17^,^18^,^19^, salt stress^20^,^21^, and wounding^7^. However, it is disadvantaged by the relatively low signal intensity, making the detection of [Ca^2+^] fluxes in individual cells and from tissues with poor sensor expression difficult^13^.

The development of Ca^2+^ biosensors that can fluoresce has complemented aequorin by allowing for detailed subcellular and tissue-level analysis of Ca^2+^ dynamics. One of the most common fluorescent biosensors are the fluorescence resonance energy transfer (FRET)-based Cameleons. FRET Cameleons are composed of two proteins, typically CFP and YFP, which are brought into close contact by the conformational change induced by the binding of Ca^2+^ to a calmodulin domain in the CFP-YFP linker region. This contact allows the transfer of energy from CFP to YFP, and the resulting change in the fluorescence of these fluorophores allows for the accurate quantification of [Ca^2+^] through the calculation of the ratio of the fluorescence signals from the two fluorophores^22^. FRET Cameleons are superior to aequorin and non-ratiometric florescent dyes, as they are less affected by the expression level of the protein^23^ and often have a greater fluorescent yield, allowing for cellular and subcellular imaging^23^. For example, FRET Cameleons have been recently used to identify long-distance Ca^2+^ signals in plants and to resolve these to the cellular level^24^,^25^,^26^.

A recent breakthrough with fluorescent GFP-based Ca^2+^ biosensors has been the development of highly sensitive single-fluorophore (single-FP) biosensors. Single-FP biosensors consist of a single circularly permutated GFP linked to a calmodulin and M13 peptide, with Ca^2+^ binding to calmodulin, resulting in a water-mediated reaction between calmodulin and GFP so as to protonate GFP and increase fluorescent yield^27^,^28^,^29^. Single-FP sensors offer several advantages over FRET Cameleons, including simpler experimental design and a potentially higher temporal resolution of imaging^30^. Although single-FP sensors cannot quantify absolute [Ca^2+^] as simply as FRET sensors, they are superior for the analysis of the temporal and spatial dynamics of Ca^2+^ signals^5^,^23^. GCaMPs are one of the best-established single-FP sensors^28^ and have undergone several revisions to enhance their fluorescent yield, dynamic range, Ca^2+^ affinity, and signal-to-noise ratios^31^,^32^,^33^,^34^. The GCaMPs have been successfully used in animal systems, such as zebrafish motor neurons^35^ and fruit fly neuromuscular junctions^34^. Random mutagenesis of GCaMP3 has resulted in additional classes of single-FP sensors, including the ultrasensitive GCaMP6^36^ and the GECOs^29^. The GECOs were recently used in *Arabidopsis thaliana *(henceforth referred to as Arabidopsis) to measure Ca^2+^ fluxes in response to ATP, chitin, and the bacterial elicitor flg22. This study also demonstrated that the R-GECO biosensor outperformed the FRET Cameleon YC3.6 in terms of maximal signal change and signal-to-noise ratio^5^.

Because of the ease of use, high fluorescent yield, and high temporal resolution that can be achieved with GCaMP biosensors, GCaMP3 was genetically encoded in Arabidopsis under the *Cauliflower mosaic virus* 35S promoter. The genetic tools available for Arabidopsis research allow for the detailed molecular analysis of the Ca^2+^ signals measured by GCaMP3. In addition, the GCaMP3 biosensor can be visualized under a fluorescence microscope rather than a costlier confocal system. This protocol allows for whole-tissue imaging, essential when conducting experiments with live biotic stresses. The experiment is designed such that detached leaves from *35S::GCaMP3* plants are floated in water, to prevent insect escape and to restrict feeding to a specific tissue. The method outlined in this paper therefore allows for the analysis of leaf Ca^2+^ dynamics during feeding by *M. persicae*, resulting in the characterization of a novel plant signaling response. This method can also be adapted to work with other biotic stresses, such as additional insect species and microbial pathogens, and with other plant tissues, such as roots.

## Protocol

### 1. Plant Preparation (Days 1 - 4)

1. On day 1, sterilize *35S::GCaMP* seeds using three 75% ethanol washes, 1 min per wash, and plate them on 100 mm^2^ square plastic plates with ¼-strength Murashige and Skoog (MS) medium (recipe: 1.1 g of Murashige and Skoog medium, 7.5 g of sucrose, 10 g of Formedium agar, and 1 L of de-ionized water)^37^.
2. Stratify the seedlings in the dark for three days at 8 °C to obtain synchronous germination.
3. On day 4 of the experiment, move the GCaMP seedlings to a controlled environment room (CER) at 23 °C, with a 16 h light and 8 h dark photoperiod.

### 2. Insect Rearing (Days 11 - 12)

1. On day 11 of the experiment, add 15 adult *M. persicae *to 5-week-old soil-grown Arabidopsis plants using a moist artist's paint brush (size 2 or 4) (grown in a CER at 22 °C with 10 h light and 14 h dark photoperiod).
2. Cage the aphids on the plant by placing clear plastic tubing (10 cm x 15 cm) around the plant and cap with a plastic lid.
3. Leave the aphid-infested plants for 24 h in a CER at 22 °C with a 16-h light and 8-h dark photoperiod.
4. On day 12 of the experiment, remove all adult *M. persicae *using a paintbrush. NOTE: Insects can be seen by eye on the plant. This gives a population of nymphs with a similar developmental age. Roughly 1 nymph per adult will be produced during this period.
5. Leave the infested plants in a lower-temperature CER at 16 °C, with an 8 h light and 16 h dark photoperiod, to prevent the nymphs from increasing in size too rapidly.

### 3. Leaf Detachment (Day 19)

1. On day 19 of the experiment, remove the *35S::GCaMP3* seedlings from the CER and detach the largest leaf from each plant using a pair of sharp scissors.
2. Using a pair of tweezers, place the detached leaf into the well of a 96-well plate containing 300 µL of distilled water, abaxial surface facing up. Repeat for additional leaves (Figure 1).

**Figure:**
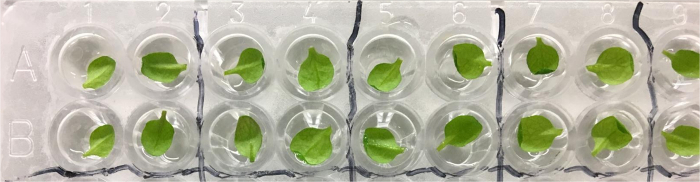

**Figure 1: *35S::GCaMP3 *Leaf Assay. **The largest leaf from each 16 day-old *35S::GCaMP3 *Arabidopsis seedling is detached and floated on 300 µL of distilled water in a 96-well plate. Photo taken the day after detachment. Please click here to view a larger version of this figure.

1. Cover the plate in clear plastic wrap and aluminum foil and leave at room temperature overnight to allow the stress of the detachment to subside.

### 4. Fluorescence Microscopy (Days 20 - 22)

1. On day 20 of the experiment, remove the 96-well plate from the aluminum foil and transfer it to a fluorescence stereomicroscope. Configure the stereo fluorescence microscope to excite GFP with a 450-490 nm light and to capture the fluorescent emission between 500 and 550 nm.
2. Collect the aged aphids from the colony established on day 11 by removing the plant from the soil and placing it in a transparent box with a lid. Keep the insects contained in the box while conducting the experiment.
3. Place the 96-well plate under the microscope. Alter the light exposure until the GCaMP3 fluorescence can be clearly visualized in the veins of the detached leaves. Keep the exposure constant for all experiments; for the current protocol, a 1-s exposure was used with a gain of 3.5 (Figure 2).
4. Adjust the magnification and zoom of the microscope such that 4 wells can be observed in one frame; for the current protocol, a 7.8X magnification and a focus of -127.833 mm was used.
5. Transfer one aphid to a detached leaf under the microscope using a moist paint brush. Leave an adjacent leaf untreated as a control, but lightly touch it with the paintbrush to mimic the touching that occurs during aphid transfer. Remember to place the plastic wrap back on top of the 96-well plate during microscopy to prevent the insect from escaping.
6. Begin the fluorescence recording of the leaves in pairs (1 aphid-treated and 1 untreated) by clicking "start experiment" in the built-in microscope software (Figure 2). Record measurements for 50 min. NOTE: For the current protocol, a time interval of 5 s between measurements was used.
7. After 50 min, stop recording by clicking on "stop experiment" and remove the aphid from the leaf. Save the fluorescence measurements as image files (*e.g.,* tagged image file format, TIFF).
8. Repeat the experiment with further pairs of leaves; imaging can be extended to image 2 pairs of leaves at once, allowing for the simultaneous imaging of 2 genotypes.

### 5. Data Collection

1. Import the image files into Fiji (Image J) and convert them to 32 bits by clicking on "Image" > "Type" > "32-bit;" this allows for the conversion of the images into heatmap videos (see step 7).
2. Discard the samples in which the aphids do not settle in one location for more than 5 min by viewing the insect movement under the microscope. NOTE: This is often the majority of samples, and up to 100 treatments may be required to find 30 samples exhibiting successful settling.
3. Set the measurement scale to pixels (or convert to mm, if known) and the time frame to the same time interval as used during the microscopy by clicking on "Image" > "Properties."
4. **Using the cursor, place a region of interest (ROI) around the area of tissue for GFP analysis.** NOTE: In the current protocol, a circular ROI 50 pixels (0.65 mm) in diameter was used at 3 locations of interest: the aphid feeding site (Fs), a systemic region on the midrib of the leaf (Sm), and a systemic region adjacent to the midrib ("lateral tissue," Sl) (Figure 2).
  1. Create the ROI by drawing an oval (use the "oval tool"), and edit the size using "Edit" > "Selection" > "Specify." See Figure 2.
5. For the untreated control, select ROIs in comparable regions of the leaf to those selected on the treated leaf (*i.e.,* the same region of the leaf as where the aphid fed on the treated leaf) (Figure 2).

**Figure:**
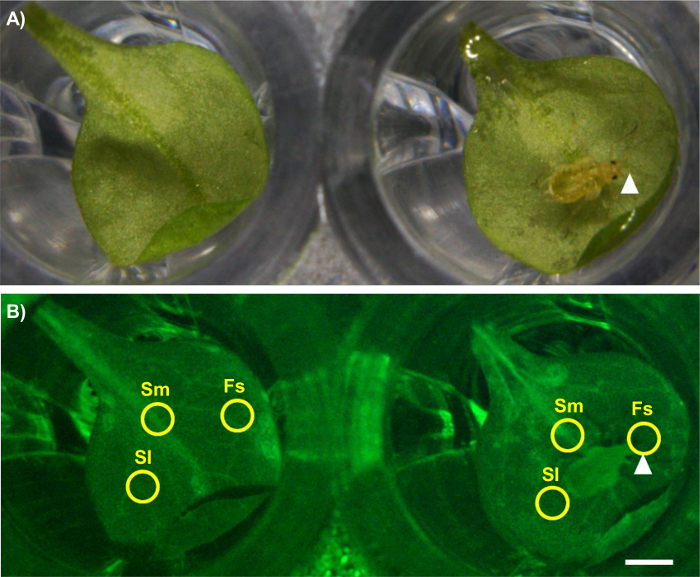

**Figure 2: Analyzing GCaMP3 Fluorescence Under the Microscope.** Left: untreated control leaf. Right: aphid-treated leaf. The aphid feeding site is indicated with an arrowhead. Scale bar = 1 mm. (**A**) Bright field image. Magnification: 7.8X, focus: -127.833 mm, exposure: 1 s. (**B**) GFP image showing the ROIs used for the quantitative analysis of fluorescence. Fs = feeding site, Sm = systemic midrib, Sl = systemic lateral tissue. Magnification: 7.8X, focus: -127.833 mm, exposure: 1 s. The GFP was excited using a 450 to 490 nm metal halide lamp, and fluorescent emission was captured between 500 and 550 nm. Please click here to view a larger version of this figure.

1. Use the Time Series Analyzer plugin by clicking "Plugins" > "Time Series Analyzer" to analyze the raw fluorescence values (F) in the ROI over time. Add the ROI of interest ("Add [t]"). Making sure that the ROI is selected, select "get average;" this will display a table of F values for each frame in that ROI.
2. Copy this data into a spreadsheet.
3. Calculate the area of the feeding site signal by first selecting the region using the "freehand selection tool." Outline the maximal GFP signal and then calculate the area of this shape by clicking "Analyze" > "Measure."
4. **Calculate the speed of the feeding site signal using the MTrackJ plugin by clicking "Plugins" > "Tracking" > "MTrackJ."**
  1. Click on the "Add" button and then click the cursor at the center of the signal when it is first visible. Click again on the edge of the signal at its point of furthest spread. Click "measure" to calculate the speed of the signal.

### 6. Data Analysis

1. Define the point of aphid settling by playing through the image frames from the microscope in Fiji. NOTE: The time of settling is the frame during which the aphid stays in a single location for 5 min or more. The duration of settling events can also be calculated from the images in Fiji.
2. Normalize the F data (ΔF/F) according to the equation ΔF/F = (F - F_0_)/F_0_, where F_0_ is the average baseline fluorescence calculated from the average of F over the first 60 frames of the recording before the aphid settled. Repeat for the untreated control.
3. Plot the normalized GFP fluorescence (ΔF/F) over time for that ROI in the treated and untreated leaf. Discard samples (both leaves) if the untreated control shows large Ca^2+^ transients (*i.e., *ΔF/F rises above 0.2).
4. Repeat until at least 20 - 30 viable samples have been analyzed per genotype.

### 7. Time-course Video Creation

1. Convert the 32-bit image files into heatmaps using the NucMed_Image LUTs plugin by clicking "Plugins" > "NucMed" > "Lookup tables." In the lookup tables menu, select "blue green red" to convert the images to heat maps.
2. Enhance the contrast of the heatmap to highlight the aphid-elicited GFP signals using "Process" > "Enhance contrast" > "Adjust saturated pixels %."
3. Add time information using the Time Stamper plugin by clicking on "Plugins" > "Time Stamper". Set the "starting time" at "0" and the "interval" based on the time interval used for the microscopy (*e.g.,* 5 s).
4. Export this video as an audio video interleaved (AVI) file. NOTE: This video can then be edited further in other software packages (*e.g.,* Microsoft Movie Maker).

## Representative Results

Figure 3 and****Figure 4****are****representative results from an experiment comparing an aphid-treated leaf with an untreated control. A highly localized increase in GFP fluorescence can be seen around the feeding site within a few minutes in the majority of samples, whilst the Ca^2+^ dynamics in the untreated control leaf stay relatively stable (Figure 3A and** 3B**). It is also possible to observe secondary increases in GFP fluorescence after the initial peak in some experiments (Figure 3B). In up to 50% of treated leaves, the aphids do not settle and the samples should be discarded. Of the samples in which settling occurs, 27% of samples do not exhibit clear increases in GFP fluorescence around the feeding site (Figure 3C and** Table 1**); therefore, 25 - 30 replicate samples should be averaged for quantitative analysis. Visualization of the area and speed of the feeding site [Ca^2+^]_cyt_ elevation should reveal a signal of around 110 µm travelling at 6 µm/s (**Table 1**). In addition, no [Ca^2+^]_cyt_ elevations should be detected systemically within the leaf upon aphid treatment, either in the systemic midrib (Figure 4A) or the systemic lateral tissue regions (Figure 4B). A representative sample of [Ca^2+^]_cyt_ dynamics over time is shown in **Video 1**. It is also possible to analyze aphid settling behavior by tracking the number and duration of individual settling events under the microscope. Representative results for these behaviors are shown in **Table 1**, showing that the aphids take around 10 min before settling, and when they do settle successfully, this lasts for 20 min on average. Therefore, the insects are settled in a single location for the entirety of the [Ca^2+^]_cyt _elevation.

**Figure:**
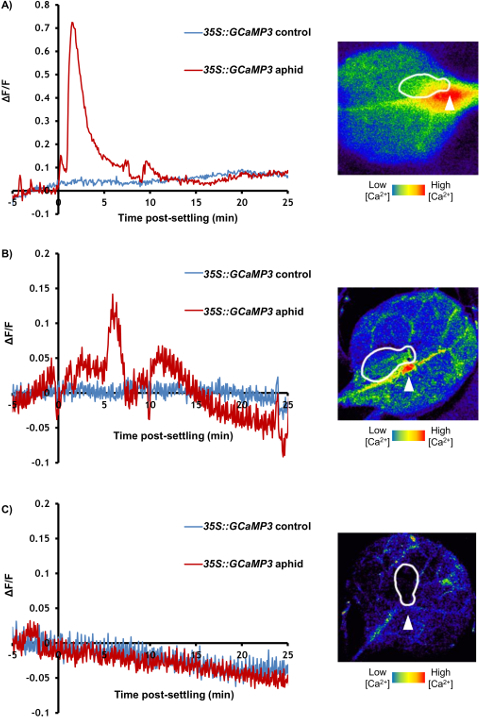

**Figure 3: GCaMP3 can be used to Detect Aphid-elicited Ca^2+^ Signals at the Feeding Site in Detached Leaves. **Left: representative traces (n = 30) of normalized GFP fluorescence (ΔF/F) around the feeding site of a *35S::GCaMP3 *leaf*. *The traces display 5 min before settling until 25 min post-settling. Control = equivalent location on an untreated *35S::GCaMP3 *leaf. Right: representative stereomicroscope image of [Ca^2+^]_cyt_ elevation seen around an aphid feeding site on a *35S::GCaMP3* leaf. The GFP fluorescence is color-coded according to the inset scale. The aphid id outlined in white and the feeding site indicated with an arrowhead. Magnification: 7.8X, focus: -127.833 mm, exposure: 1 s. The GFP was excited using a 450 to 490 nm metal halide lamp, and the fluorescent emission was captured between 500 and 550 nm. (**A**) An example of a large aphid-induced [Ca^2+^]_cyt _elevation. (**B**) An example of an average aphid-induced [Ca^2+^]_cyt _elevation. (**C**) An example of no aphid-induced [Ca^2+^]_cyt _elevation. Please click here to view a larger version of this figure.

**Table:** 

**Parameter**	**Average (± SEM)**
**[Ca^2+^]_cyt_ elevation**	
Percentage of samples displaying a [Ca^2+^]_cyt_ elevation	73%
Speed of wave front ^a^	5.9 µm/s (± 0.6)
Maximum area of spread	110 µm^2^ (± 18)
**Aphid behaviour**	
Number of settles (>5 min)	2 (± 0.1)
Total number of settles (all durations)	3.8 (± 0.4)
Time settled for imaging ^b^	20 min (± 2)
Time until first settle ^c^	11 min (± 1.4)
Percentage of total time spent settled	62% (± 3)

**Table 1: [Ca^2+^]_cyt  _Elevation and Aphid Behavior Parameters during *35S::GCaMP3 *Imaging. **Parameters calculated from viable samples in which settling of >5 min occurred.****(**A**) Speed of the visible signal from the point of initiation to the furthest point of spread. (**B**) Duration of the initial settling events used for the analysis of fluorescence. (**C**) Length of time between the beginning of imaging and the first aphid settle. [Ca^2+^]_cyt _elevation data previously submitted to The Plant Cell (current status: initial QC).

**Figure:**
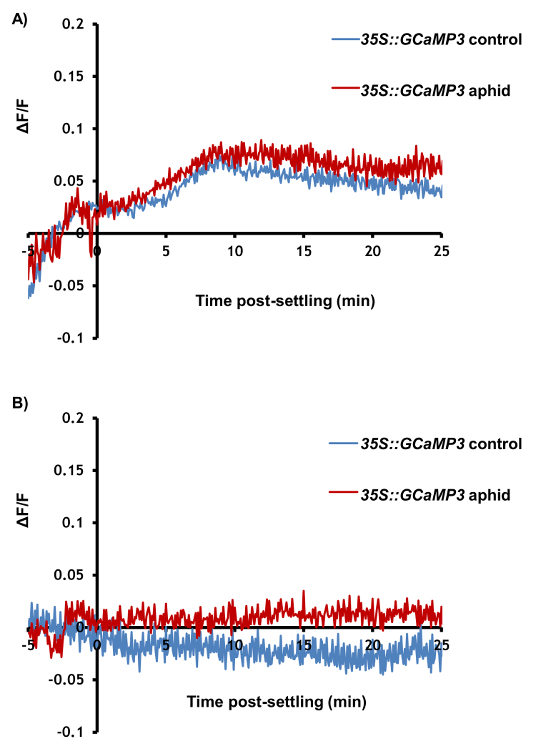

**Figure 4: GCaMP3 Cannot Detect Aphid-elicited Ca^2+^ Signals Systemic to the Feeding Site. **Representative traces (n = 30) of normalized GFP fluorescence (ΔF/F) in locations systemic to the aphid feeding site in *35S::GCaMP3 leaves. *The traces display 5 min before settling until 25 min post-settling. Control = equivalent location on an untreated *35S::GCaMP3 *leaf. (**A**) ΔF/F in the systemic midrib region. (**B**) ΔF/F in the systemic lateral tissue region. Please click here to view a larger version of this figure.


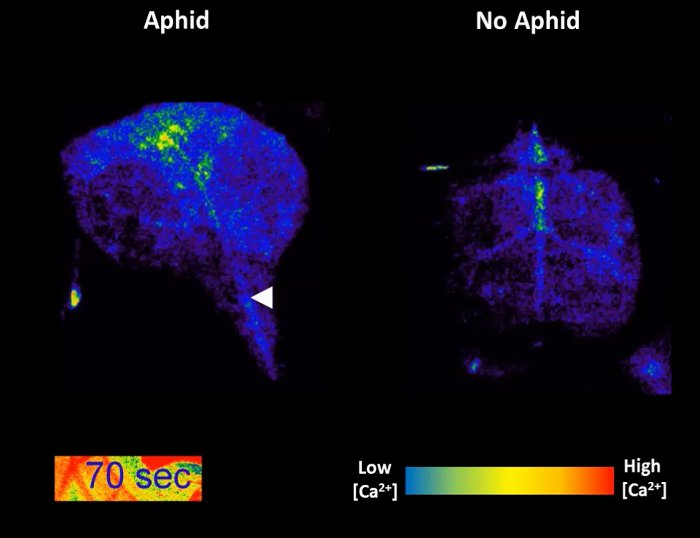
**Video 1: GCaMP3 Florescence Over time as an Aphid Feeds. **The GFP fluorescence is color-coded according to the inset scale. The location of the aphid feeding site is indicated with an arrowhead. Left: *35S::GCaMP3* leaf exposed to an *M. persicae *adult. Right: A *35S::GCaMP3* no-aphid control leaf. Please click here to view this video. (Right-click to download.)

## Discussion

The method described in this paper allows for the real-time analysis of plant Ca^2+^ signaling during a biotic stress such as insect feeding. It demonstrates that one of the first plant responses to such threats is a localized [Ca^2+^]_cyt_ elevation around the feeding site of the insect. Through the use of mutants, this method will allow for the the molecular and physiological characterization of such signals, which was not previously possible. A critical step in this protocol is to ensure that the detached leaves are not excessively disturbed during the detachment process (step 3.2) or when transferring insects to the leaves (step 4.5). Given that the current protocol provides a relative measurement of [Ca^2+^]_cyt _rather than an absolute concentration, it is vital that the microscope settings are kept constant throughout the experiment. There is also the potential for human bias during the selection of ROIs and the analysis of the data, and as such, it is recommended that the experiments are conducted double-blind.

There are several significant advantages of measuring [Ca^2+^]_cyt _during biotic stress with this protocol. First, the use of a single fluorophore with a high fluorescent yield allows the imaging to be conducted on a stereomicroscope, which is less costly than using a confocal microscope. The use of a single fluorophore also makes data collection and analysis simple, as there is just one measurement to record. In addition, the use of a stereomicroscope allows for the imaging of entire leaves, which is essential given that many biotic interactions, including plant-aphid interactions, occur on a large spatial scale. The high temporal resolution of image capture possible with GCaMP3, based on the rapid disassociation of Ca^2+^ from the sensor after binding^23^,^30^ and the high florescent yield, allows for measurements to be taken up to every 5 s. Furthermore, the leaf assay prevents the escape of the insect, a key limiting step to conducting such experiments on whole plants^ (in preparation)^. The detached leaves also ensure that the insect feeds from a pre-defined location, allowing for the analysis of Ca^2+^ dynamics before, during, and after feeding. This protocol also ensures that leaves of similar developmental stages are used for analysis.

The main disadvantage of this protocol originates from the use of a non-ratiometric biosensor. With single-FP biosensors, variation in GFP emission may result from experimental variables other than [Ca^2+^]_cyt_, such as changes in cellular pH, motion, or the expression level of the biosensor. These issues are not encountered with FRET Cameleons during FRET, as the transfer of energy from CFP to YFP only occurs upon Ca^2+^ binding. Other conditions that alter the fluorescent properties of the individual fluorophores are unlikely to mimic the opposing changes in intensity of CFP and YFP, and the ratiometric calculation that is used inherently normalizes the measurements for many of these other optical artifacts^23^,^30^. This makes estimations of absolute [Ca^2+^]_cyt _more reliable with FRET Cameleons. Consequently, GCaMP3 is best used as a biosensor to measure relative [Ca^2+^]_cyt_, although it is still sufficient to detect and characterize biological phenomena in plants^5^^,(in preparation)^. Therefore, it is essential to use controls to show that the observed effect is due to Ca^2+^, including Ca^2+^-related genetic mutants^(in preparation)^ or pharmacological Ca^2+^ channel inhibitors such as La^3+^. Importantly, single-FP biosensors typically display a greater fluorescent yield and greater dynamic range (*i.e., *an increase in florescence upon Ca^2+^ binding) than FRET Cameleons^23^, which makes GCaMP more suited to tissue-level imaging, while FRET Cameleons are a useful tool for cellular imaging with a confocal microscope^5^,^25^.

During the execution of this protocol, it is possible that some issues will arise that require troubleshooting. For example, it is recommended that samples in which the control (untreated) leaf displays large [Ca^2+^]_cyt _elevations are discarded (step 6.3). Such transients are most likely the result of stress induced by the microscopy. Indeed, blue light is known to elicit Ca^2+^ signals^38^,^39^,^40^,^41^, and the high-intensity light might also result in temperature and osmotic stresses, both of which also elicit [Ca^2+^]_cyt_ elevations^21^,^25^,^42^. Consequently, to reduce such stresses, it is important to conduct the experiment in a well-ventilated and temperature-controlled room and to avoid unnecessarily long exposure times. It is also important to not disrupt the leaves excessively during detachment or during the microscopy to prevent touch-elicited [Ca^2+^]_cyt_ elevations^43^,^44^,^45^. Issues may also be encountered with insect settling. With *M. persicae*, the insects do not settle on the leaves in several samples. This could be a result of wound-elicited defense in the detached leaves^46^,^47^, or the disturbance of the insects by the blue light. Indeed, vision in *M. persicae *is governed by three photoreceptors, including one with a peak sensitivity of 490 nm^48^. Reducing the microscopy exposure and handling the aphids with care might reduce distress and encourage settling.

The protocol outlined in the current paper gives new insights onto the molecular understanding of plant-insect interactions and the plant response to biotic stress. It allows for the visualization of one of the first plant responses to insect feeding and facilitates further investigations through the use of the considerable Arabidopsis genetic resources available. In addition, this protocol allows for the use of live organisms, as opposed to extracts^49^ or elicitors^50^. In the future, this technique could be applied to other biotic stresses, such as additional insect species, nematodes, or microbial pathogens, as well as to abiotic stresses. The GCaMP3 microscopy can also be modified to image other plant tissues, alternative ROIs on the leaf, or even whole plants. Furthermore, there is the potential for the biosensor to be genetically encoded in additional plant species. Consequently, the protocol outlined in this paper has the potential to undercover the molecular basis of Ca^2+^ signaling in a range of novel biotic interactions between plants and other species.

## Disclosures

The authors have no conflicts of interest to declare.
